# Ceftriaxone Suppresses Group II Metabotropic Glutamate Receptor Expression Contributing to Reversal of Recognition Memory Deficits of Amyloid Precursor Protein/Presenilin 1 AD Mice

**DOI:** 10.3389/fnins.2022.905403

**Published:** 2022-07-04

**Authors:** ShuJuan Fan, Li Li, LiRong Liu, He Li, XiaoHui Xian, WenBin Li

**Affiliations:** ^1^Department of Pathophysiology, Neuroscience Research Center, Hebei Medical University, Shijiazhuang, China; ^2^Department of Central Laboratory, The Second Hospital of Hebei Medical University, Shijiazhuang, China

**Keywords:** ceftriaxone, Group II mGluRs, GLT-1, APP/PS1 mice, SNAP-25

## Abstract

Group II metabotropic glutamate receptors (Group II mGluRs) are the peri-synaptic receptor of glutamatergic neurons and negatively regulate glutamate release from presynaptic neurons. Glutamate in the synaptic cleft is mainly taken into astrocytes by glutamate transporter-1 (GLT-1), which is primarily expressed in astrocytes. Increasing evidence showed that inhibiting or suppressing the activation of Group II mGluRs would contribute to the improvement of learning and memory deficits in Alzheimer’s disease (AD) animal models. Ceftriaxone (Cef) has been reported to alleviate the spatial memory deficits in AD model mice by improving GLT-1-related clearance and metabolism of glutamate. Therefore, the present study further investigates the improving effect of Cef on recognition memory deficits and the involvement of Group II mGluRs in the process using the APP/PS1 AD mouse model. Novel object recognition tests showed that the Cef treatment significantly improved the recognition memory deficits of the AD mice. The Western blot and immunohistochemistry analysis showed that the Cef treatment significantly suppressed the upregulation of Group II mGluRs expression in APP/PS1 AD mice. The above suppression effect of Cef was blocked by dihydrokainic acid, an inhibitor of GLT-1 uptake activity. Furthermore, the Cef treatment significantly restored the downregulation in the downstream molecules of Group II mGluRs activation, including the expression of PKA and phosphorylated SNAP-25 in the APP/PS1 AD mice. The Cef treatment had no effect on the content of Aβ_40_ and Aβ_42_ in the hippocampus of APP/PS1 AD mice. The above results suggested that the suppression of Group II mGluRs contributed to the Cef-induced reversal of the recognition memory deficits in APP/PS1 AD mice.

## Introduction

Glutamate is a major type of excitatory neurotransmitters in the central nervous system and plays an important role in learning, memory, and cognition (Danbolt, 2001). Group II metabotropic glutamate receptors (Group II mGluRs) are the peri-synaptic receptor of glutamatergic neurons and mainly distribute in the presynaptic membrane (Swanson et al., 2005; Niswender and Conn, 2010). The activation of Group II mGluRs negatively regulates the presynaptic glutamate release and synaptic transmission between glutamatergic neurons (Kintscher et al., 2012; Wang et al., 2012). Series of studies have indicated that the soluble Aβ significantly impairs the synaptic transmission between glutamatergic neurons by activating Group II mGluRs (Caraci et al., 2011). For instance, the incubation of brain slices with the agonist of Group II mGluRs deteriorated the Aβ-induced excitability reduction of both presynaptic and postsynaptic neurons (Chin et al., 2007; Li et al., 2009). *Vice versa*, inhibiting the function of Group II mGluRs increased the synaptic transmission between glutamatergic neurons (Tyszkiewicz and Yan, 2005) and significantly alleviated the Aβ-induced cognitive dysfunction of Alzheimer’s disease (AD) mice (Kim et al., 2014). These findings indicated that the over-activation of Group II mGluRs was involved in the Aβ-induced neuronal toxicity, as well resulted in a reduced glutamate release and weakened synaptic transmission between glutamatergic neurons (Hoxha et al., 2012; Mitew et al., 2013; Poirel et al., 2018). Therefore, inhibiting or suppressing the activation of Group II mGluRs would contribute to improve the learning and memory deficits of AD mice.

Group II mGluRs are activated by excessive accumulation of glutamate in the peri-synaptic region resulted from glutamate spillover from synaptic clefts, which occurred with the increased glutamate concentration in the synaptic clefts (Pinheiro and Mulle, 2008; Sheffler et al., 2011). Therefore, reducing the glutamate concentration in synaptic clefts timely would be an effective way to inhibit the excessive activation of Group II mGluRs in AD. Glutamate in the synaptic cleft is taken into astrocytes mainly by glutamate transporters. Since the glial glutamate transporter-1 (GLT-1) takes the majority of glutamate (account for up to 70%) from synaptic clefts and plays an important role in glutamate homeostasis in the peri-synaptic region (Anderson and Swanson, 2000), modulating the expression and/or function of GLT-1 to increase the glutamate uptake and decrease the glutamate spillover would inhibit the over-activation of Group II mGluRs and contribute to improve the learning, memory, and cognitive deficits in AD.

Rothstein et al. (2005) reported that Ceftriaxone (Cef), a kind of beta-lactam antibiotics, could significantly and selectively upregulate the expression of GLT-1 *in vitro* and *in vivo* studies (Liu et al., 2012; Krzyzanowska et al., 2016). In AD studies, it was found that Cef could improve the spatial working memory in AD mice by upregulating the expression of GLT-1 and GLT-1-related glutamate metabolism (Zumkehr et al., 2015; Fan et al., 2018). Therefore, the present study was undertaken to further investigate the effect of Cef on recognition memory impairments of AD mice and the role of group II mGluRs and the related downstream molecules, including protein kinase A (PKA) and synaptosomal-associated protein 25 kDa (SNAP-25), in the process. The elucidation of these issues would provide new evidence for figuring out the mechanisms of Cef improving learning and memory disorders in AD mice, and provide new clues for the study of AD prevention and treatment.

## Materials and Methods

### Animals and Grouping

C57BL/6J wild type (WT) mice and heterozygous APPswe/PS1dE9 (APP/PS1) transgenic AD model mice at the same age with WT mice were used in the study. This type of AD model mice is bred in a C57BL/6J genetic background and over-expresses human amyloid precursor protein (APP) with the Swedish (K594M/N595L) mutation and presenilin 1 (PS1) deleted in exon 9 (Duyckaerts et al., 2008). The model starts producing amyloid plaques from 5 months old, with peak production occurring at 12 months old. The model exhibits pathological manifestations consistent with AD (Webster et al., 2014).

The study consisted of the following four groups: WT group, APP/PS1 group, Cef group (according to the dose of Cef, the mice were further divided into 100, 200, and 300 mg/Kg subgroups), and DHK (dihydrokainic acid) + Cef group. In the Cef group, the APP/PS1 AD mice were intraperitoneally injected with Cef (Roche, Switzerland, dissolved in normal saline at a concentration of 285 mg/ml) once a day for continuous 14 days. Normal saline was injected to WT and APP/PS1 mice in the same volume and protocols with Cef in WT and APP/PS1 groups. In the DHK + Cef group (DHK, Sigma, United States, 10 mg/Kg), DHK was administrated by intraperitoneal injection 30 min before each Cef injection. The other treatments were the same with the Cef group. After the completion of the administrations, the mice in all groups received novel object recognition test to evaluate their recognition memory. To avoid systemic errors, the test was repeated in 6-month and 7-month-old mice. Mice in all groups were decapitated under deep isoflurane anesthesia after the behavioral test. The brains of 6-month-old group mice were used in the following subsequent assays.

A total of 160 mice were used in the study, and the numbers of mice in each group for each test are shown in Table 1. All animals were provided with food and water *ad libitum* and were housed on a 12-h light/dark schedule (25°C). All treatments were conducted in accordance with the ARRIVE guidelines (Animal Research: Reporting of *In Vivo* Experiments), and were approved by the Committee of Ethics on Animal Experiments of Hebei Medical University. All efforts were made to minimize the suffering and numbers of the animals.

**TABLE 1:** The number of mice in each group and assay.

Groups	NOR-7 months	NOR-6 Months	WB	IHC/IF	ELISA
WT	*n* = 18	*n* = 12	*n* = 4	*n* = 3	*n* = 5
APP/PS1	*n* = 25	*n* = 23	*n* = 4	*n* = 3	*n* = 5
Cef-100 mg/Kg	*n* = 12	*n* = 10	/	/	/
Cef-200 mg/Kg	*n* = 15	*n* = 14	*n* = 4	*n* = 3	*n* = 5
Cef-300 mg/Kg	*n* = 12	*n* = 11	*n* = 4	/	*n* = 5
DHK + Cef-200 mg/Kg	/	*n* = 8	*n* = 4	/	/

*The mice used for WB, IHC/IF, and Elisa assays were randomly selected from the 6-month-old mice subjected with the NOR test. Cef, Ceftriaxone; DHK, dihydrokainic acid; NOR, novel object recognition; WB, western-blot; IHC, immunohistochemistry; IF, Immunofluorescence. The symbol “/” means no mice were used in this assay.*

### Novel Object Recognition Test

This test was performed according to previous reports (Olszewski et al., 2017; Wu et al., 2018). Briefly, an open field apparatus was used, which composed of 4 polyvinyl chloride plastic box (40 cm wide, 40 cm long, and 30 cm high) placed in a sound-attenuated cabinet. Illumination of the cabinets was provided by a LED light (30 Lux) mounted on the ceiling of the cabinet, and background noise at 25 dB was supplied by a ventilation fan in the cabinet. An overhead video camera coupled to a computer was used to track animal movements.

**FIGURE 1:**
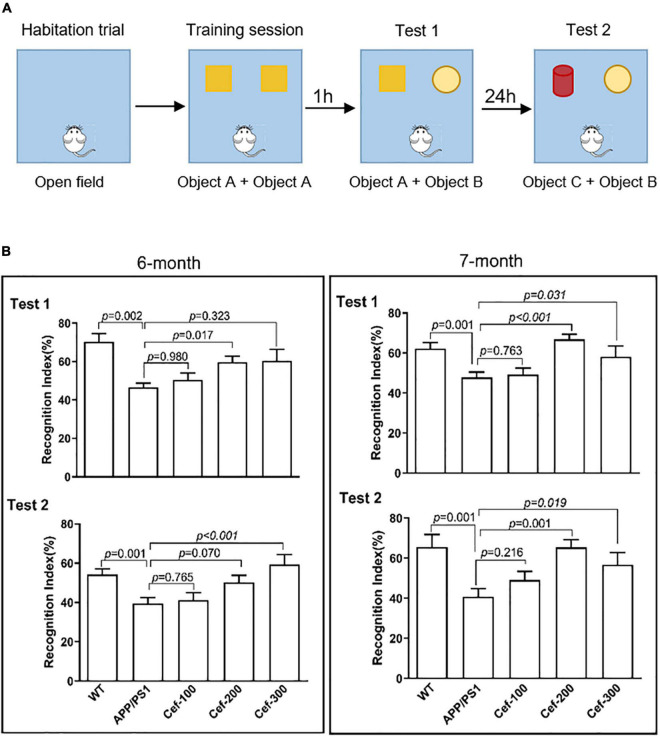
Cef improves recognition memory deficits in 6-month and 7-month-old APP/PS1 mice. **(A)** Shows the protocol of novel object recognition test. **(B)** Shows the recognition index of the tests in 6-month and 7-month-old APP/PS1 mice in each group. The number of mice for each group are shown in Table 1. WT, wild type; Cef, ceftriaxone. The italic values upon the bars are statistical *P* values.

The protocol of the test consisted of habituation, training, and testing trials (Figure 1A). During the habituation trial, mice were individually habituated to the open field arena for 10 min in the absence of objects. No data were collected during the habituation. The training session was performed in the next day, and the mouse was placed in the arena and allowed to freely explore the arena in the presence of two identical objects (object A, orange wooden cubes with side of 3 cm) for 5 min. Testing trial consisted of test 1 and test 2. The test 1 was performed at 1 h after the training session to evaluate the short-term recognition memory. The test 2 was performed at 24 h after the training session to evaluate the long-term recognition memory. At the above time points, the mouse was re-introduced into the open-field arena that contained a familiar object A and a novel object B (wooden hemisphere with diameter of 4 cm) for the test 1, and a familiar object B and a novel object C (a red wooden cylinder with diameter 3 cm and height 5 cm) for the test 2. The mouse was scored as exploring an object when its head oriented toward the object within a distance of 2 cm or when its nose touched the object. Sitting on or going around the objects was not considered exploratory. The mouse was allowed in the open-field arena for 5 min and the exploration time for the familiar (TF) or the novel object (TN) during the test trial was recorded. The recognition memory was defined by the recognition index, which was calculated according to the following formula: [TN/(TN + TF)] × 100%. The mouse was returned to its home cage after each session, the arena and the objects of the apparatus were cleaned thoroughly between trials to ensure the absence of olfactory cues.

### Western Blot Analysis

The expressions of mGluR2 (the primary subtype of Group II mGluRs which mainly distributed in the presynaptic terminals and negatively regulates glutamate release) (Jin et al., 2018), PKA, SNAP25, and phosphorylated-SNAP25 in the hippocampus of the mice were assayed by the Western blot analysis. The hippocampus of the mice was homogenized using tissue lysis buffer supplemented with 1 mM PMSF and protease inhibitors (Roche, Switzerland). The protein concentrations in the supernatants were determined by bicinchoninic acid and assayed by Synergy-HT microplate reader (BioTek, Gene Company Limited, United States). The loading buffer, including the reducing agent mercaptoethanol, was added to the supernatants, and then, the supernatant was boiled in boiling water to make protein denaturation. Twenty-microgram proteins were separated by the SDS-PAGE electrophoresis and transferred onto polyvinylidene fluoride membranes (Millipore, Billerica, MA, United States). After blocking in 5% (w/v) commercial skim milk for 1 h at 37°C, the membranes were incubated overnight at 4°C with monoclonal primary antibodies derived from rabbits against mGluR2 (Abcam, United Kingdom, Cat: ab150387, Lot: GR105860-10, 1:1000 dilution), PKA (Abcam, United Kingdom, Cat: ab32390, Lot: GR31302-44, 1:1000 dilution), SNAP25 (Abcam, United Kingdom, Cat: ab5666, Lot: GR3255764-5, 1:1000 dilution), and phosphorylated-SNAP25 (Abcam, United Kingdom, Cat: ab169871, Lot: GR128291-10, 1:1000 dilution). After washing with TPBS, the membranes were incubated with second antibody (Proteintech, Rosemont, IL, United States, Cat: SA00001-2, HRP labeled-goat anti-rabbit IgG, Lot: 20000373, 1:2000 dilution) at room temperature for 1 h. The GAPDH (for PKA) or β-actin (for phosphorylated-SNAP25 and total-SNAP25) was used as a loading control. The immunoreaction was developed by enhanced chemiluminescence high sensitive substrate (Lot: 130531, Monad Biotech Co., Ltd., China) and detected using Amersham Imager 600 (GE Healthcare UK Limited, United Kingdom). The bands of the immunoblot were analyzed by densitometry using image analyzing software (Alpha Imager, United States). The ratios of integral optical density (IOD) between aimed proteins and β-actin/GAPDH from the same homogenate were used to quantitatively present the relative expression of the aimed proteins.

### Immunohistochemistry and Immunofluorescence

To evaluate the relative expression levels and distribution of mGluR2 (the reason why assay the expression of mGluR2 is the same as the above mentioned), immunohistochemistry was performed on paraffin-embedded sections. After deparaffinization by xylene, hydration in descending alcohol and incubation with 3% (v/v) H_2_O_2_ for 30 min to eliminate the endogenous peroxidase and with 10% (w/v) goat serum to block the non-specific antigen, the sections were incubated with the primary antibody against mGluR2 (monoclonal antibody derived from mouse, Lot: RC2173551, Thermo Fisher Scientific, Waltham, MA, United States, 1:300 dilution) overnight at 4°C. Then, the sections were incubated at 37°C for 1 h with the secondary antibody (HRP-labeled-goat anti-mouse IgG, Lot: 140012, KPL, Ocracoke, NC, United States 1:600). The immunoreaction was visualized using diaminobenzidine (ZSGB, Beijing, China). Because of the diffuse distribution of mGluR2, we selected the area in the peak of the arch of the CA1 subfield in 400× magnification for the quantitative analysis of the immunoreactive density. The mean optical densities of selected areas were measured by the ImageJ software (NIH Image, United States) to quantify the density of immunoreactivity.

To detect the presynaptic distribution of mGluR2 in the CA1 hippocampus, we performed double immunofluorescence labeling of mGluR2 and synapsin I on paraffin sections. After deparaffinization with xylene and hydration in a series of decreasing concentrations of alcohol, and antigen repairment using microwave, the sections were incubated with 10% (w/v) goat serum, to block the non-specific antigens. Then, the sections were incubated overnight at 4°C with the mixture of primary antibodies against mGluR2 (the same antibody used in immunohistochemistry staining) and Synapsin I (Abcam, United Kingdom, Cat: ab254033, polyclonal antibody derived from rabbit, Lot: GR3242284, 1:300 dilution). In the next day, the sections were incubated with the corresponding second antibody for mGluR2 (FITC-conjugated anti-mouse IgG derived from donkey, Cat: SA00003-9, Proteintech, Rosemont, IL, United States, 1:500 dilution) and Synapsin I (TRITC-conjugated anti-rabbit IgG derived from goat, Cat: SA00007-2, Proteintech, Rosemont, IL, United States, 1:500 dilution). The fluorescent images were excited at 488 and 594 nm, respectively, and captured using a laser confocal microscope (FV1200, Olympus, Japan).

### ELISA

The extraction of soluble and insoluble Aβ species of the hippocampal homogenates was referred to the previous reports (Luo et al., 2020). The content of soluble and insoluble Aβ_1–40_ and Aβ_1–42_ in the homogenates was detected using enzyme linked immunosorbent assay (ELISA) kits according to the manufacturer’s instructions. The OD value was read at 450 nm using the Synergy-HT microplate reader (BioTek, Gene Company Limited, United States).

### Statistics

All statistical analyses were performed using SPSS for Windows 21.0 software (SPSS Inc., Chicago, IL, United States). All data were presented with mean ± SEM and analyzed using One-way ANOVA. Different significances between groups were tested using the LSD test, except the recognition index in the test 1 of novel object recognition of 6-month-old mice, the IOD value of Western-blot of mGluR2 and the level of insoluble Aβ42, for which, the Dunnett T3 test was used because of variance uneven. The statistical significance was assumed if *p* < 0.05.

## Results

### Ceftriaxone Improved the Recognition Memory Deficits of Amyloid Precursor Protein/Presenilin 1 AD Mice

The novel object recognition test in 6-month-old mice showed that the recognition index of APP/PS1 mice in both test 1 and test 2 was obviously declined compared with the wild-type mice (Figure 1B, 6 months) [test 1: *F*_(4_,_65)_ = 7.193, *p* < 0.001; test 2: *F*_(4_,_62)_ = 5.595, *p* = 0.001, ANOVA]. The Cef treatment at dose of 200 or 300 mg/Kg significantly reversed the decline in the recognition index of APP/PS1 mice in either test 1 or test 2 (Figure 1B in test 1, *p* = 0.017 for Cef 200 mg/Kg group; in test 2, *p* < 0.001 for Cef 300 mg/Kg group), although no significant effect was observed at low dose of 100 mg/Kg (*p* = 0.980 in test 1 and *p* = 0.765 in test 2). The performance of the 7-month-old group mice in the novel object recognition test, including test 1 and test 2 (Figure 1B, 7 months) [test 1: *F*_(4_,_77)_ = 6.502, *p* < 0.001; test 2: *F*_(4_,_55)_ = 4.883, *p* = 0.002, ANOVA], was similar with that of 6-month-old mice group. The results indicated that the Cef treatment significantly improved the deficits of recognition memory in APP/PS1 AD mice.

### Ceftriaxone Suppressed the Upregulation in the Expression of mGluR2 in Amyloid Precursor Protein/Presenilin 1 AD Mice

**FIGURE 2:**
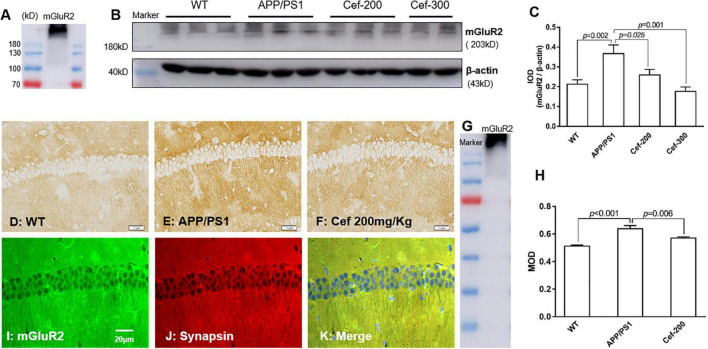
Cef suppresses the expression of mGluR2 in protein level in the hippocampus of 6 month-old APP/PS1 mice. **(A)** Is an mGluR2 band to indicate the expression of dimeric mGluR2 (230 kD). **(B)** Is a representative immunoblot of Western blot in each group, and **(C)** is the quantitative presentation of the immunoblot with integral optical density (IOD). **(D–F)** Are representative photomicrographs of immunohistochemistry staining in each group and the scale bar on them is 20 μm. **(G)** Is a western blot band of mGluR2 to indicate the immunohistochemistry stain is specific for mGluR2. **(H)** Is the quantitative presentation of the immunohistochemistry staining with mean optical density (MOD) in each group. **(I–K)** Are representative photomicrographs of double immunofluorescent labeling staining of mGluR2 and synapsin in WT mice. The number of mice for each test is shown in Table 1. mGluR2, metabotropic glutamate receptor 2. The italic values upon the bars are statistical *P* values.

The Western-blot analysis (Figure 2A) showed an immunoreactive band at molecular weight of 230 kD, which indicated the expression of dimeric mGluR2. This is consistent with the previous report that dimeric mGluR2 is an active state of mGluR2. It was shown by the Western blot analysis (Figures 2B,C) that there has a basal expression of mGluR2 in the hippocampus of the wild-type mice. Compared with the wild-type mice, the immuno-bands were larger and denser in the APP/PS1 AD mice (Figure 2B), and IOD of the immuno-bands was significantly increased (Figure 2C, *p* = 0.002) [*F*_(3_,_18)_ = 6.384, *p* = 0.004, ANOVA], indicating the increased expression of mGluR2. Cef treatment in the dose of 200 and 300 mg/kg significantly inhibited the expression of mGluR2 in APP/PS1 mice represented with weaken immuno-bands (Figure 2B) and decreased IOD of the immunoreactivity (Figure 2C, *p* = 0.025 and *p* = 0.001, respectively) in the Cef treatment group compared with APP/PS1 AD mice. The immunohistochemistry showed similar results that mGluR2 immunoreactivity increased in the CA1 hippocampus in APP/PS1 mice and Cef treatment suppressed the increase (Figures 2D–H) [*F*_(2_,_10)_ = 22.100, *p* < 0.001, ANOVA]. Furthermore, double immunolabeling staining of mGluR2 and synapsin showed that the mGluR2 is mainly expressed on neuronal terminals in the hippocampus (Figures 2I–K). The above results suggested that the Cef treatment suppressed the upregulation of mGluR2 expression in the neuronal terminals in the hippocampus of APP/PS1 mice.

### Dihydrokainic Acid Blocked Ceftriaxone- Induced Reversal on Recognition Memory Deficits and Suppression on the mGluR2 Expression in Amyloid Precursor Protein/Presenilin 1 AD Mice

**FIGURE 3:**
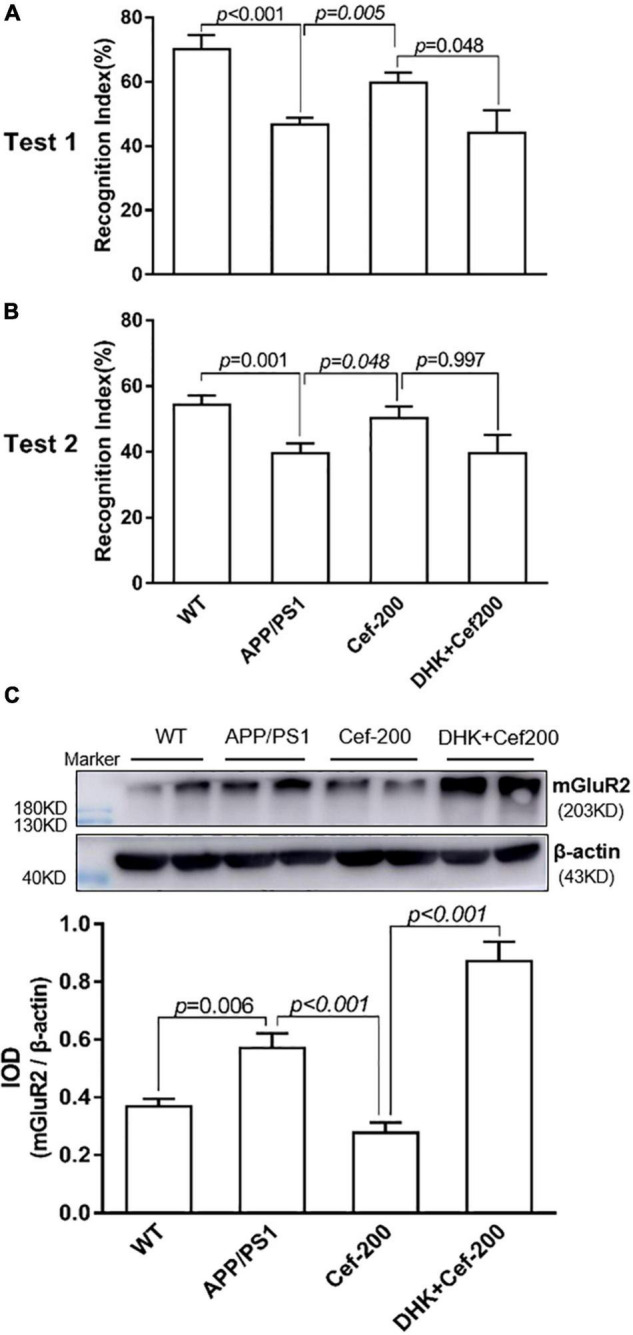
DHK blocks Cef-induced improvement on recognition memory deficits and suppression on the mGluR2 protein expression in APP/PS1 mice. The histograms of **(A,B)** show the recognition index in test 1 **(A)** and test 2 **(B)** of the novel object recognition test. **(C)** Shows the expression of mGluR2 assayed by the Western blot analysis. The upper is a representative immunoblot and the lower is the quantitative presentation of the immunoblot with IOD. The number of mice for each test is shown in Table 1. The italic values upon the bars are statistical *P* values.

To determine whether Cef-induced reversal on recognition memory deficits and suppression on the mGluR2 upregulation in APP/PS1 AD mice were associated with GLT-1 or not, we further investigated the effect of DHK, a selective inhibitor of GLT-1, on the recognition memory and mGluR2 expression after Cef treatment in APP/PS1 mice. In the novel object recognition test, the Cef-induced reversal on recognition index in APP/PS1 AD mice was inhibited after the administration of DHK in DHK + Cef group compared with Cef group (Figures 3A,B) [test 1: *F*_(3_,_53)_ = 10.503, *p* < 0.001; test 2: *F*_(3_,_58)_ = 4.860, *p* = 0.004, ANOVA]. Meanwhile, the suppression on mGluR2 expression in APP/PS1 AD mice induced by Cef was also reversed by the administration of DHK (Figure 3C) [*F*_(3_,_20)_ = 30.966, *p* < 0.001, ANOVA]. The results suggested that Cef-induced reversals on the recognition memory deficits and suppression on the mGluR2 upregulation in APP/PS1 AD mice were dependent on GLT-1.

### Ceftriaxone Increased the Expression of Protein Kinase A and Phosphorylated Synaptosomal-Associated Protein 25 kDa in Amyloid Precursor Protein/Presenilin 1 AD Mice

**FIGURE 4:**
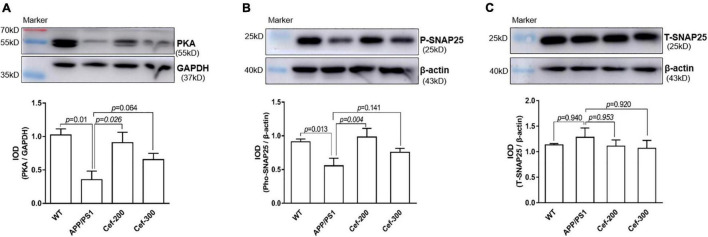
Cef upregulates the expression of PKA and phosphorylated SNAP-25 in the hippocampus of APP/PS1 mice. The upper in each of **(A–C)** is a representative immunoblot of Western blot and analysis, and the lower is the quantitative presentation of the immunoblot with IOD. The number of mice for each test is shown in Table 1. PKA, protein kinase A; P-SNAP25, phosphorylated synaptosomal-associated protein 25 kDa; T-SNAP25, total synaptosomal-associated protein 25 kDa. The italic values upon the bars are statistical *P* values.

The Western blot analysis showed that there was an obviously decrease in the expression of PKA and phosphorylated SNAP25 in APP/PS1 AD mice compared with wild-type mice (Figures 4A,B, *p* = 0.01 and *p* = 0.013, respectively) [for PKA: *F*_(3_,_16)_ = 6.002, *p* = 0.006, ANOVA; for phosphorylated SNAP-25: *F*_(3_,_20)_ = 4.180, *p* = 0.019, ANOVA]. After Cef treatment in APP/PS1 mice, the downregulation of PKA and phosphorylated SNAP-25 was significantly alleviated, represented with larger and denser immune-reactive bands and increased IOD of the bands compared with APP/PS1 group (Figures 4A,B, *p* = 0.026 and *p* = 0.004, respectively). There was no significantly difference in the expression of total SNAP-25 between groups (Figure 4C) [*F*_(3_,_12)_ = 0.474, *p* = 0.706, ANOVA]. The above results suggested that Cef significantly increased the activation of PKA and SNAP-25 to against their downregulation in APP/PS1 AD mice.

### Ceftriaxone Had No Effect on Aβ Levels in the Hippocampus of Amyloid Precursor Protein/Presenilin 1 Mice

**FIGURE 5:**
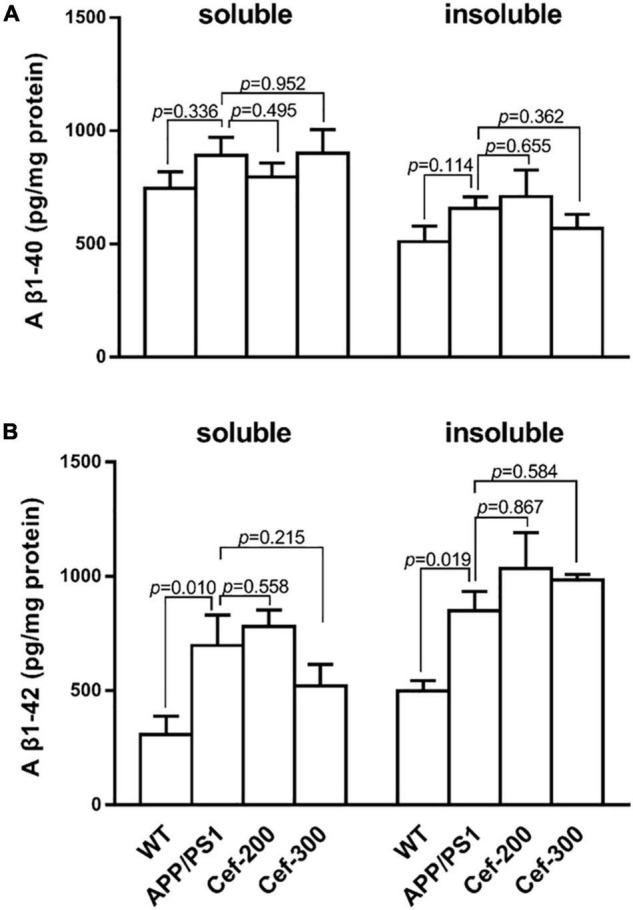
Cef has no effect on Aβ levels in the hippocampus of APP/PS1 mice. **(A)** Is the assay of soluble and insoluble Aβ_1–40_ levels in each group, and **(B)** is the soluble and insoluble Aβ_1–42_ levels. The number of mice for each test is shown in Table 1. The italic values upon the bars are statistical *P* values.

ELISA assay (Figure 5) showed that the levels of soluble and insoluble Aβ peptides, including Aβ_1–40_ (Figure 5A) and Aβ_1–42_ (Figure 5B) in the hippocampus, were lower in the wild-type mice at 6-month old age. In APP/PS1 mice, the burden of either soluble or insoluble Aβ_1–42_ significant increased compared with wild-type mice (Figure 5B, *p* = 0.010 and *p* = 0.019, respectively) [*F*_(3_,_20)_ = 4.626, *p* = 0.013, ANOVA], while soluble or insoluble Aβ_1–40_ levels had no difference (Figure 5A, *p* = 0.336 and *p* = 0.114, respectively) [*F*_(3_,_27)_ = 0.623, *p* = 0.606, ANOVA]. The Cef treatment had no effect on the Aβ levels.

## Discussion

Many studies have shown that modulating the activation of Group II mGluRs would be a beneficial and effective strategy in several animal models, such as the nicotine or alcohol addiction (Dhanya et al., 2011; Cross et al., 2018; Windisch and Czachowski, 2018), schizophrenia (Xing et al., 2018), epilepsy (Ali et al., 2016), and ischemia brain damage animal models (Bratek et al., 2018). In the present study, we found that the expression of mGluR2 was upregulated in APP/PS1 AD mice. This finding is consistent with and supported by previous reports that Aβ could significantly upregulate Group II mGluRs (Caraci et al., 2011). It is well-known that Group II mGluRs negatively regulates glutamate release as a neurotransmitter in glutamatergic neurons. Thus, the upregulation in the expression of Group II mGluRs would lead to decrease of the release of glutamate neurotransmitter, and then weaken synaptic transmission between glutamatergic neurons in the APP/PS1 AD mice. It was reported that the glutamate released from presynaptic neurons is reduced with progressed cognitive deficits and follows the weakened synaptic transmission between glutamatergic neurons in the prefrontal cortex of AD patients and AD animal models (Hoxha et al., 2012; Mitew et al., 2013; Poirel et al., 2018). Thus, the upregulation in the expression of Group II mGluRs might be an important factor involved in the pathogenesis of AD. Furthermore, we showed that the Cef treatment significantly suppressed the upregulation in the expression of mGluR2 in APP/PS1 AD mice, and especially this suppression induced by Cef was accompanied by the reversal in the recognition memory of APP/PS1 AD mice. So, it is deducible that the suppression of mGluR2 induced by Cef would weaken the inhibition on glutamate release from presynaptic terminals and increase glutamate release as a neurotransmitter, which would facilitate the synaptic transmission and contribute to the reversal of cognitive deficits in the AD mice.

To illustrate the suggestion above mentioned, we further investigated the changes in the expression of PKA and SNAP-25, which are the downstream molecules of mGluR2 activation and associated with the glutamate release from presynaptic membranes. The results indicated that in APP/PS1 AD mice, the expression of PKA and phosphorylated SNAP25 was decreased accompanied with the upregulation in the expression of mGluR2 although the total SNAP-25 was unchanged. It was reported that intracellular Aβ oligomers directly inhibited the exocytosis of glutamate by impairing SNAP-25-mediated SNARE complex formation (Yang et al., 2015). PKA, as a downstream molecule of mGluR2, is responsible for the phosphorylation of subsequent downstream molecules, including SNAP-25. The phosphorylated SNAP-25 by PKA promotes the fusion of the vesicular and presynaptic membranes and plays an important role in regulating the exocytosis of neurotransmitters. So, it might be supposed that in AD model mice, the abnormally activated mGluR2 inhibits cAMP-PKA signal, reduces the phosphorylation of SNAP-25 (Arnsten et al., 2012; Gao et al., 2012), hampers the vesicular fusion with pre-terminal membrane and hence reduces the vesicular glutamate release. Notably, after the Cef treatment in the present study, the downregulated expression of PKA and phosphorylated SNAP25 was rescued following the restoration of mGluR2 expression. These findings indicated that PKA and phosphorylated SNAP25, as the downstream signals of Group II mGluRs, were involved in the Cef-induced reversal of cognitive deficits in the APP/PS1 mice. Therefore, it might be suggested that suppressing the excessive activation of Group II mGluRs by Cef or other medications or methods might be an effective strategy or target for the study in the prevention and therapy of AD. The previous studies showed that pharmacologically inhibiting the activation of the Group II mGluRs could increase the synaptic transmission between glutamatergic neurons in an AD mouse model (Oliveira et al., 2008). This report supports our findings and inference.

Many studies have indicated that Cef could significantly and selectively increase the expression and uptake activity of GLT-1 *in vitro* and *in vivo* studies, and this upregulation has shown a beneficial effect in many disease models, such as cerebral neurons in animal models of stroke, oxygen glucose deprivation, and motor neuron degeneration (Rothstein et al., 2005; Liu et al., 2012; Krzyzanowska et al., 2016). To determine whether the suppression of Cef on the expression of mGluR2 is dependent on GLT-1, we investigated the effect of DHK, a specific function inhibitor of GLT-1, on the Cef-induced suppression of mGluR2 expression in the APP/PS1 mice. The results showed that the Cef-induced suppression of mGluR2 expression in the APP/PS1 mice was blocked by DHK. Simultaneously, the Cef-induced reversal of the recognition memory deficits was also blocked by DHK. Azar et al. (2009) reported a similar finding that Cef blocked the mGluR-dependent long-term depression at the mossy fiber-CA3 hippocampal synapse, while the blocked long-term depression was rescued by DHK. Our recent study has shown that Cef could upregulate the GLT-1 expression and uptake activity in APP/PS1 AD mice. Thus, the above findings after administration of DHK suggested that it is the upregulation of the GLT-1 expression and uptake activity, by which Cef suppressed the mGluR2 expression and improved the recognition memory in APP/PS1 mice.

It might be interesting to outline the mechanisms that how Cef suppresses the mGluR2 expression and improves the cognitive deficits in APP/PS1 AD mice by upregulation of GLT-1. As known, GLT-1 takes most glutamate released from presynaptic neurons into peri-synaptic astrocytes (Anderson and Swanson, 2000). In AD, GLT-1 expression and its uptake activity for glutamate are impaired, which lead to a decreased glutamate uptake, and then cause glutamate spillover. The glutamate spillover over activates mGluR2 resulting in inhibition of glutamate release and decrease in the quality of new released glutamate in the synaptic cleft, which decrease the “signal to noise ratio” of the synaptic transmission (Jin et al., 2018) and cause cognitive impairments. Cef, by inducing GLT-1 upregulation, could play a crucial role of “one stone, two birds” in keeping the homeostasis of glutamate in the peri-synaptic area and in the reproduction of glutamate as neurotransmitter. On the one hand, Cef-induced GLT-1 upregulation could timely remove glutamate from synaptic clefts and decrease the glutamate concentration in and spillover of glutamate from the synaptic clefts. These effects then decrease the activation of mGluR2 (Upreti et al., 2013) and its inhibition on the release of glutamate from pre-terminals as neurotransmitter. On the other hand, Cef could promote glutamate transport into astrocytes by GLT-1 upregulation. This could provide more substrate and then enhance the glutamate–glutamine cycle and glutamate production as neurotransmitter (Fan et al., 2021), which plays a crucial role in facilitating synaptic transmission between glutamatergic neurons because over 70% of glutamate released from presynaptic neurons comes from the glutamate–glutamine cycle between the peri-synaptic astrocytes and neuronal terminals (Tani et al., 2014). The above effects of Cef in the two aspects by upregulating GLT-1 might facilitate the synaptic transmission and contribute to the improvement of cognitive deficits in the APP/PS1 AD mice in the present study.

The previous studies found that in transgenic AD mice, activation of Group II mGluR2 triggers Aβ_42_ production and release from isolated intact nerve terminals (Kim et al., 2010), and results in an increase of the soluble Aβ levels in the brain of AD mice (Ulus and Wurtman, 1997). We found in the present study that the levels of soluble and insoluble Aβ_1–40_ and Aβ_1–42_ were significantly increased, combined the increased mGluR2 expression in APP/PS1 mice. This finding is consistent with the above previous reports. However, Cef treatment did not accordingly decrease the levels of the Aβ oligomers in the hippocampus of APP/PS1 mice despite that Cef decreased the mGluR2 expression. This result is similar with the report by Zumkehr et al. (2015). The reason for the likely paradox result might be related to the magnitude of the mGluR2 suppression. In the present study, Cef’s effects on mGluR2 expression were dependent on GLT-1 signaling, as DHK prevents Cef from reducing mGluR2 expression. The suppression of the mGluR2 by these manners might be not enough to suppress the production and release of Aβ from neuronal terminals.

## Conclusion

The Cef treatment, by upregulating GLT-1 expression, suppresses the expression of mGluR2 contributing to the reversal of the recognition memory deficits of APP/PS1 AD mice and the upregulation in the expression of PKA and SNAP-25, at least partly, is involved in the process.

## Data Availability Statement

The original contributions presented in this study are included in the article/supplementary material, further inquiries can be directed to the corresponding authors.

## Ethics Statement

The animal study was reviewed and approved by the Committee of Ethics on Animal Experiments of Hebei Medical University. Written informed consent was obtained from the owners for the participation of their animals in this study.

## Author Contributions

SF: immunofluorescence, Western-blot assay, and the original manuscript preparation. LL: immunofluorescence assay and the analysis of experimental data. LRL: novel object recognition test and Western-blot assay. HL: the raising and breeding of the transgenic mice used in the study. XX: reviewing and editing the manuscript. WL: the whole experimental design and guidance, reviewing and polishing the manuscripts. All authors contributed to the article and approved the submitted version.

## Conflict of Interest

The authors declare that the research was conducted in the absence of any commercial or financial relationships that could be construed as a potential conflict of interest.

## Publisher’s Note

All claims expressed in this article are solely those of the authors and do not necessarily represent those of their affiliated organizations, or those of the publisher, the editors and the reviewers. Any product that may be evaluated in this article, or claim that may be made by its manufacturer, is not guaranteed or endorsed by the publisher.
